# Energy saving and emission reduction fiscal policy and corporate green technology innovation

**DOI:** 10.3389/fpsyg.2022.1056038

**Published:** 2022-12-06

**Authors:** Huan Jin, Jing Yang, Yinying Chen

**Affiliations:** ^1^School of Business, East China University of Science and Technology, Shanghai, China; ^2^School of Economics and Business Administration, Chongqing University, Chongqing, China

**Keywords:** energy saving and emission reduction fiscal policy, green technology innovation, organizational change, organizational effectiveness, sustainable development

## Abstract

With the increasing prominence of resource and environmental issues, countries around the world are paying more and more attention to the concept of sustainable development. Under this concept, China started to implement a pilot project of “National Comprehensive Demonstration City of Energy Saving and Emission Reduction Fiscal Policy” in 2011 to protect resources and environment through green and low-carbon development. This paper aims to investigate whether and how the pilot policy induces corporate green technology innovation. Based on the data on Chinese listed firms from 2008 to 2019 and the relevant theories of economics, management and organizational psychology, we find that the pilot policy can promote corporate green technology innovation. This indicates that the pilot policy, as an external force, will encourage firms to improve their adaptability through green technology innovation which is one type of organizational change, thus improving their organizational effectiveness. The heterogeneity analyses reveal that the promotion effect of the pilot policy on green innovation is stronger among firms in high-carbon industries, firms in the mature stage and firms that are not state-owned. The mechanism tests find that the credit allocation effect and innovation compensation effect generated by the pilot policy are the key channels to promote green technology innovation. In addition to enriching the research on the evaluation of the effects of the pilot policy, our paper also expands the literature on organizational psychology and organizational change from the perspective of corporate green innovation, offers practical implications for the low-carbon transition of manufacturing industries under the emission peak and carbon neutrality targets, and provides insights for other emerging economies to achieve better resource and environmental protection through the energy saving and emission reduction fiscal policy.

## Introduction

Nowadays, resource and environmental problems are becoming increasingly serious, posing a critical challenge to the sustainable development of mankind. In 1987, the World Commission on Environment and Development (WCED) first raised the concept of sustainable development, emphasizing the importance of reconciling economic development with resource conservation and environmental protection. Since then, countries around the world have gradually incorporated the concept of sustainable development into their plans. As latecomers of economic development, emerging economies find it difficult to balance the protection of resources and the environment while pursuing economic development. In order to save resources and protect the environment, governments usually implement environmental policies to solve the problems of resources and the environment. Enterprises are modern forms of organizations that aim at profitability, and they constantly exchange materials, energy and information with the external environment in order to constantly reform and develop (Schein, 2011). On the one hand, strict environmental regulations increase the cost of pollution control for enterprises, which may become an external pressure for organizational change and is not conducive to enterprise competitiveness; on the other hand, it may also force enterprises to carry out organizational innovation and management innovation, which becomes an external incentive for organizational change and enhances enterprise competitiveness. Therefore, it is of theoretical and practical significance to study how emerging economies can achieve sustainable development through environmental policies.

The traditional neoclassical view is that while environmental regulations can solve pollution problems, it can also increase firms’ production costs and reduce profitability, leading to a decline in firm productivity and economic performance (Barbera and Mcconnell, 1990; Jorgenson and Wilcoxen, 1990). In the 1990s, Porter and Linde (1995) argued that from a dynamic point of view, environmental regulations can stimulate technological and organizational innovation and promote rational allocation of production resources, thus generating an “innovation compensation effect” that can compensate for the cost of regulations, which is known as the “Porter hypothesis.” Since then, scholars have developed a rich verification around the Porter hypothesis (Gray and Shadbegian, 2003; Wagner, 2005; Hamamoto, 2006; Kneller and Manderson, 2012; Bergek and Berggren, 2014; Wang and Liu, 2014). Currently, a growing literature supports the Porter hypothesis and suggests that technological innovation is the key to improving energy efficiency, environmental protection and green development (Chakraborty and Chatterjee, 2017; Xu and Cui, 2020; Xu et al., 2022). However, financial bottlenecks are a major obstacle to green technology innovation in enterprises. Organizational psychology argues that companies need to overcome psychological inertia, improve psychological tolerance and psychological adaptability when undertaking organizational changes like innovation (Christensen and Bower, 1996; Rindova et al., 2010). Innovation is characterized by high cost, high risk, and uncertain payback period. Given the high risk and investments in green technology innovation projects, whether a company chooses to invest in green technology innovation depends on the management’s motivation and willingness to innovate. If the management is in a good frame of mind regarding expected future returns but lacks financial support, then it is likely that the management will not choose green technology innovation activities. Therefore, financial bottlenecks are a major impediment to corporate green technology innovation, which makes compensation mechanisms for green technology innovation by financial institutions and capital markets increasingly important (Bento and Fontes, 2015). Expanding capital markets and broadening financing channels are necessary guarantees for green cycles and sustainable development in emerging economies (Zeng et al., 2017).

Since the implementation of the Reform and Opening up Policy, China has achieved rapid development and become the world’s second largest economy and one of the typical representatives of emerging economies. In order to promote its own enterprises to establish a green, recycling, low-carbon development concept and to give full play to the green fiscal funds to guide the role of energy saving and emission reduction, in 2011, the National Development and Reform Commission of the People’s Republic of China issued the Notice on Comprehensive Demonstration of Energy Saving and Emission Reduction Fiscal Policy, setting six objectives which include the achievement of industrial low-carbonization, reduction of major pollutants, and scaling up of renewable energy use, and subsequently further expanding the scope of the pilot project. However, there are a very few existing studies evaluating the effects of this policy. At present, only Lin and Zhu (2019) and Xu et al. (2022), from the macro perspective of urban eco-efficiency and carbon emissions, have evaluated the carbon emission reduction effects and sustainable development effects of the energy saving and emission reduction fiscal policy. However, the existing research lacks empirical evidence from micro enterprises level. Therefore, based on the perspective of green technology innovation of enterprises, this paper adopts the difference-in-differences (DID) method to systematically evaluate the energy saving and emission reduction fiscal policy and tries to answer the following questions: First, can the policy promote corporate green technology innovation? Second, is there a Porter effect? If there is, what is the mechanism by which the pilot policy promotes green technology innovation in enterprises? Lastly, are there any variations in policy effects in different regions, industries and for different types of enterprises? The answers to the above questions can not only enrich and complement the research in areas related to the Porter hypothesis, but also provide empirical support for emerging economies to guide the green and low-carbon transition of enterprises through green fiscal policies.

Therefore, our paper takes the pilot construction of National Comprehensive Demonstration City of Energy Saving and Emission Reduction Fiscal Policy implemented and gradually promoted in China since 2011 as the entry point, manually screens out green patent data of Chinese A-share listed companies from 2008 to 2019 based on the Green List of International Patent Classification launched by WIPO and the international patent classification numbers. Based on this data and the relevant theories of economics, management and organizational psychology, we systematically explore the green technology innovation effect of China’s energy saving and emission reduction fiscal policy. We find that the pilot policy can induce corporate green technology innovation. This suggests that as the government and other stakeholders pay more attention to environmental protection, the pilot policy, as an external force, will promote firms to improve their adaptability through green technology innovation which is one type of organizational change, thus improving their organizational effectiveness. Furthermore, the green technology innovation effect of the policy starts to appear in the fourth year after the construction of the demonstration city and gradually increases year by year. The mechanism analyses show that the pilot policy promotes corporate green technology innovation mainly through the funding allocation effect and innovation compensation effect. Moreover, the green technology innovation effect of the policy has obvious industry and enterprise heterogeneity, and the induced effect of green technology innovation is more pronounced when the enterprises are within high-carbon industries, when the enterprises are in mature stage, and when the enterprises are not state-owned.

Compared to previous studies, our paper makes several contributions. First, based on the relevant theories of economics, management and organizational psychology, our paper examines the implementation effects of the energy saving and emission reduction fiscal policy from the perspective of corporate green technology innovation for the first time, providing empirical evidence at the micro-firm level for the research in this area, thus enriching the study of the economic consequences of the energy saving and emission reduction fiscal policy. Second, our study documents that the energy saving and emission reduction fiscal policy can alleviate the psychological barriers faced by the management in making organizational change (i.e., corporate green innovation) through the funding allocation effect, which extends the literature on organizational psychology and organizational change from the perspective of corporate green innovation. Third, the in-depth research on the differential impacts of enterprise ownership, enterprise life cycle and industrial characteristics on inducing green technology innovation will provide directions for the orderly promotion and precise implementation of the pilot policy in the future. Fourth, our paper provides empirical evidence from emerging economies for the studies related to the Porter hypothesis, which provides a useful addition to this field. At last, this study could also provide useful insights for other emerging economies to guide the green and low-carbon transition of enterprises through green fiscal policies.

The rest of our paper is arranged as follows: the second part introduces the policy background, the third part presents the related literature and hypothesis development, the fourth part introduces the research design of our study, the fifth part shows the empirical results of the benchmark analysis, the sixth part further examines the mechanism and heterogeneity of the pilot policy inducing green technology innovation, and the last part contains conclusions and policy implications.

## Policy background

In order to establish the concept of green, recycling and low-carbon development, accelerate the construction of energy saving and emission reduction work led by the government, dominated by enterprises, effectively driven by the market and participated by the whole society, in June 2011, the Ministry of Finance and the Development and Reform Commission jointly issued the Notice on the Comprehensive Demonstration of Energy Saving and Emission Reduction Fiscal Policy, which identifies eight cities (e.g., Beijing, Hangzhou, Shenzhen, Chongqing, etc.) to take the lead in launching comprehensive pilot for the energy saving and emission reduction fiscal policy.

In October 2013, the second batch of comprehensive demonstration cities were added, including Shijiazhuang, Tangshan City, Qiqihaer City, Tongling City, and so on; in 2014, 12 new cities (e.g., Urumqi City, Tianjin, Linfen City, Xuzhou City, Liaocheng City, etc.) were added as the third batch of comprehensive demonstration cities. These three batches of cities cover 27 provinces in China, and each city has its own characteristics in terms of economic development, industrial structure, resource endowment and environmental carrying capacity, which can reflect the differentiation and representativeness of the demonstration cities.

The policy explicitly states that the construction of demonstration city needs to complete six tasks, the main one of which is to achieve industrial low-carbonization. It also requires to ① Eliminate backward production capacity and equipment, support key enterprises to implement energy-saving technology transformation, and vigorously promote the application of advanced energy-saving and environmental protection technologies and equipment; ② Improve the threshold of access to high-energy-consuming and high-emission industries and the level of energy consumption limits for major energy-consuming products; ③ Accelerate the development of strategic emerging industries and advanced service industries, and optimize the industrial structure. In specific practice, the demonstration city construction promotes the low-carbon transformation of the city by means of financial funding guidance. During the demonstration period, the central government will give financial incentives to the typical demonstration projects declared and filed by the demonstration cities, from which the municipalities directly under the central government, provincial capitals and other cities will be awarded 600, 500, and 400 million yuan of green financial funds per year, respectively. The demonstration cities decide how to use the comprehensive incentive funds, and the central government will only be responsible for the record management of the relevant projects.

## Literature review and research hypothesis

### Literature review

The relationship between environmental regulations and technological innovation has always been a research hotspot in the field of environmental economics. This paper presents a literature review from two aspects: the impact of environmental regulations on green technology innovation and the research related to energy saving and emission reduction policies.

The research on green innovation originated in the 1990s, most of which mainly refers to green technology innovation. The definition of green technology innovation usually varies according to the research topic. Rennings (2000) defines green technology innovation as a series of value creation activities that can generate new products or new processes, and contributes to reducing environmental burdens and achieving ecological sustainability purposes. The green technology innovation defined by the World Intellectual Property Organization (WIPO) covers the widest range, including environmentally relevant pollutant disposal and technologies related to climate change mitigation, and contains the patent classification numbers of all related technologies. Regarding the impact of environmental policies on green technology innovation, the existing research mainly focuses on the Porter hypothesis. Based on theoretical analyses and case studies, Porter and Linde (1995) believe that designing reasonable environmental regulations can help promote corporate technological innovation and organizational innovation, which will not only improve environmental performance, but also partially, sometimes even completely offset the additional regulatory costs. Jaffe and Palmer (1997) first distinguish between the strong Porter hypothesis and the weak Porter hypothesis.

Currently, most studies support the weak Porter hypothesis. Using the cost of pollution control as a measure of the intensity of environmental regulations, Hamamoto (2006) empirically tests the impact of environmental regulations on technological innovation in manufacturing, and finds that the Porter effect does exist: the stronger the environmental regulation, the higher the level of innovation characterized by R&D expenditure. Bergek and Berggren (2014) argue that well-designed environmental regulations can force energy-intensive firms to engage in environmentally friendly green technology innovation activities. Some scholars also put forward the opposite view, arguing that the pressure of environmental regulations can adversely affect enterprises’ production and operation, and thus inhibit their R&D innovation. For example, Kneller and Manderson (2012), using a sample of enterprises in United Kingdom manufacturing industry, find that environmental regulations lead to higher compliance costs for enterprises, which forces resources to shift from traditional production to pollution control, resulting in some crowding-out effect on corporate investment in innovation. The cost of regulations is particularly pronounced in resource-intensive industries (Gray and Shadbegian, 2003).

The research on energy saving and emission reduction policies mainly includes two directions. Early literature uses the total emissions of pollutants and the rate of compliance with emission standards as measures of energy saving and emission reduction by local governments to examine the impact of environmental regulations on efficiency gains or productivity growth of enterprises (Domazlicky and Weber, 2004). Such indicators equate the results of pollution control with environmental regulation itself, which leads to a serious endogeneity between environmental regulations and pollution control. Therefore, it cannot be simply assumed that the intensity of environmental regulations is high if the pollutant emissions are low or the compliance rate is high. Johnstone et al. (2010) and Chakraborty and Chatterjee (2017) examine the impact of renewable energy policies on firms’ patent applications and R&D investment from a patent perspective, but they fail to strictly distinguish between green technology innovation and other non-environmental technology innovation. In recent years, most of the literature adopts demonstration policies to examine the environmental and economic effects of energy saving and carbon reduction policy with the help of causal inference methods. Using the establishment of a national comprehensive demonstration city for energy saving and emission reduction fiscal policy as a quasi-experiment, Lin and Zhu (2019) construct a DID model to examine the impact of the policy on urban sustainable development. The results show that the eco-efficiency of Chinese cities has been effectively improved during the implementation period of the policy, but there is a time lag of at least 3 years for the policy effects. In the context of China’s emission peak and carbon neutrality targets, Xu et al. (2022) construct a DID model using the energy saving and emission reduction fiscal policy to examine the impact of the policy on carbon emission reduction in 284 cities in China. They find that the policy significantly reduces urban CO_2_ emissions. Xu and Cui (2020) shift their research perspective from the mid-macro level to the firm level and investigate the impact of China’s low-carbon city pilot policy on corporate green technology innovation using a DID method. The results show that China’s low-carbon city pilot policy can induce corporate green technology innovation to a certain extent, especially for high carbon emission industries and non-state enterprises.

### Research hypothesis

According to organizational psychology theory (Schein, 2011), as an important organization, a company consists of many interacting, interdependent and mutually influencing subsystems. Therefore, when making organizational change in a company (e.g., green technology innovation), it is essential to take the individual changes of key members as the starting point and the important intermediary (Schein, 2011), especially to overcome the psychological barriers of the management which includes changing its attitudes and values (Gioia and Chittipeddi, 1991), eliminating potential threats, overcoming psychological inertia, and improving psychological tolerance and psychological adaptability (Christensen and Bower, 1996; Rindova et al., 2010). Unlike conventional production operations and general investment, corporate green technology innovation is a change characterized by high investment, high cost and high risk, which constitutes a major psychological barrier to the management in carrying out corporate green technology innovation and thus affects managers’ motivation for conducting corporate green technology innovation. Without sufficient motivations, organizational change will not happen (Schein, 2011). Therefore, how to overcome the psychological barriers of the management and motivate the management to carry out corporate green technology innovation becomes the core issue for the green technology innovation of companies.

Energy saving and emission reduction fiscal policy can alleviate the psychological barriers faced by the management in making corporate green technology innovation through the funding allocation effect which includes credit support and government subsidies, and thus promotes firms’ green technology innovation. On the one hand, the central government will give comprehensive financial incentives to the demonstration cities included in the assessment of energy saving and emission reduction through financial allocation. Moreover, the central government also provides an extra 20% incentives for the cities that have achieved their energy saving and emission reduction targets and gained excellent assessment results. This provides sufficient incentives for the enterprises in the demonstration cities to conduct green technology innovation.

On the other hand, the financial funds like credit support and government subsidies can alleviate corporate financial constraints, which will greatly mitigate the management’s concerns about decisions of green technology innovation and overcome the psychological barriers of the management when conducting green technology innovation. Under the guidance of green financial funds, the firms in the demonstration cities can obtain finance support more easily and thus may have a stronger motivation to conduct green technology innovation. Therefore, we expect that the energy saving and emission reduction fiscal policy will provide a new financing channel for firms in the demonstration cities, and thus have a positive impact on the firms’ green technology innovation. We hence propose our first hypothesis as follows:

*Hypothesis 1:* Energy saving and emission reduction fiscal policy will promote firms’ green technology innovation.

Whether a firm carries out innovation after obtaining financial funds depends on two considerations. On the one hand, well-designed environmental policies can generate a Porter effect, which helps to motivate enterprises to increase their R&D investment (Porter and Linde, 1995). In the process of building demonstration cities, local governments use both financial incentives and target constraints to jointly promote energy saving and emission reduction within the demonstration cities. If the policy is well designed, this combination of policy tools will encourage enterprises to increase their R&D investment. In addition, the demonstration cities also set up special funds for energy saving and emission reduction to encourage enterprises to carry out low-carbon technology research and development through direct incentives and other means.

On the other hand, if enterprises only choose to purchase large-scale emission reduction equipment rather than independent research and development after obtaining fiscal support, such inefficient investment will lead to a failure of the fiscal policy. Jin et al. (2022) empirically evaluate the policy effect of China Green Finance Reform and Innovation Pilot Zone and find that although the establishment of the pilot zone increases the credit funds of non-heavily polluting enterprises, it fails to promote their green technology innovation, because enterprises would choose policy arbitrage in order to obtain more green credit. Similarly, if the assessment and supervision mechanisms in the demonstration cities are not in place, the green funds of enterprises may shift from productive areas to non-productive activities, thus crowding out their R&D investment. Therefore, it is uncertain whether enterprises invest in R&D activities after obtaining fiscal support. We hence propose that:

*Hypothesis 2a*: Energy saving and emission reduction fiscal policy promotes firms’ green technology innovation through R&D innovation compensation effect.

*Hypothesis 2b*: Energy saving and emission reduction fiscal policy fails to produce innovation compensation effect, which is detrimental to firms’ green technology innovation.

## Research design

### Sample and data

The initial sample in our paper includes all the listed companies in manufacturing industry from 2008 to 2019. Firms with missing data and firms receiving a Special Treatment status are excluded. The data used in our paper include three categories. First, the financial data at the enterprise level are obtained from the China Securities Market and Accounting Research database (CSMAR). Second, the city-level data are from the China City Statistical Yearbook and the Economy Prediction System (EPS) database. Third, we manually collect corporate green patents based on the Green List of International Patent Classification issued by the World Intellectual Property Organization (WIPO) and the names of listed companies. Moreover, in order to further reflect the types and value of green patents, we divide green patents into green invention patents and green utility patents. At last, all continuous variables are winsorized at the first and 99th percentiles.

### Indicator construction

The purpose of our research is to examine the effect of the energy saving and emission reduction fiscal policy on firms’ green technology innovation. Using the International Patent Classification (IPC) numbers in the Green List of International Patent Classification issued by the WIPO and the names of listed firms as keywords, we manually collect firms’ green patents from the official website of the State Intellectual Property Office. The number of green patent applications is used as a proxy for corporate green technology innovation. We use firms’ green patent applications as the dependent variable for the following two reasons: Firstly, compared to R&D inputs, patents may more naturally reflect the outputs of firms’ green technology innovation activities, and they have a clear technical categorization which can reflect the value and contributions of innovation activities; Secondly, considering the long time taken from patent application to grant, using patent application data may assess the impact of the energy saving and emission reduction fiscal policy on green technology innovation in a more effective way. Following Xu and Cui (2020), we exclude the missing values (*Null*) of green patent data, and only keep the green patent data of enterprises with application records in the current year. We use three indicators, total green patents (*EnvirPat*), green invention patents (*EnvirInvPat*) and green utility patents (*EnvirUtyPat*), to measure green patent applications and take logarithms of them.^1^ In the robustness test, we also use the number of green patents granted (*PatGrant*), the number of green invention patents granted (*InvPatGrant*) and the number of green utility patents granted (*UtyPatGrant*) as alternative measures to ensure the reliability of the regression results.

In addition, we control for other factors that may influence firms’ green technology innovation. ① The scale of the firm (*scale*), which is an important factor influencing firm innovation (Bu et al., 2020). The larger the firm, the more likely it is to have sufficient funds to conduct green technology innovation activities. We use the logarithm of total assets at the end of the year to measure *scale*. ② Leverage ratio (*lev*). The more leveraged the firm is, the greater its debt risk tends to be, which is not conducive to green technology innovation. Therefore, we use the ratio of a firm’s total liabilities to total assets at the end of the year to measure *lev*. ③ The older the firm, the more mature and conservative the firm might be, which may lead the firm to make only minor modifications to their original technologies and products rather than invest more in green technology innovation (Kueng et al., 2014). Hence, we control for *age* in our model. The indicator *age* is computed as the natural logarithm of the firm’s age. ④ The ratio of fixed assets (*fixasset*) is measured as the ratio of net fixed assets to total assets. The higher the ratio, the more difficult it may be for companies to transform and innovate green. ⑤ Management shareholding ratio (*share*) is computed as the ratio of management shareholding to total shareholding. ⑥ Tobin’s Q (*tobinQ*). We use the sum of the market value of shareholders’ equity and the book value of liabilities divided by the book value of total assets at year-end and then take the logarithm to measure *tobinQ*. ⑦ Two positions in one (*one*). The indicator *one* takes the value of 1 if the chairman and the Chief Executive Officer (CEO) are the same person, and 0 otherwise. Table 1 shows the descriptive statistics of the main variables.

**Table 1:** Descriptive statistics.

Variables	*N*	Mean	S.D.	Min	Max
*EnvirPat*	16,873	0.320	0.760	0.000	6.440
*EnvirInvPat*	16,873	0.220	0.610	0.000	6.110
*EnvirUtyPat*	16,873	0.190	0.540	0.000	5.350
*PatGrant*	14,893	0.270	0.660	0.000	5.600
*InvPatGrant*	14,893	0.120	0.420	0.000	4.680
*UtyPatGrant*	14,893	0.200	0.560	0.000	5.140
*DID*	16,873	0.210	0.400	0.000	0.937
*scale*	16,873	21.85	1.200	19.32	26.06
*lev*	16,873	0.400	0.200	0.050	0.970
*age*	16,868	2.760	0.340	1.790	3.500
*fixasset*	16,873	0.240	0.140	0.001	0.730
*share*	16,424	0.160	0.220	0.000	0.690
*tobinQ*	16,301	0.600	0.460	−0.120	2.100
*one*	16,715	0.300	0.460	0.000	1.000

*N* indicates the number of the sample observations. Mean, Min, and Max denote variables’ mean value, minimum, and maximum, respectively. S.D. indicates the standard deviation of variables.

### Empirical model

In order to examine the impact of the energy saving and emission reduction fiscal policy on corporate green technology innovation, we use the establishment of National Comprehensive Demonstration City of Energy Saving and Emission Reduction Fiscal Policy as a quasi-experiment and construct an asymptotic difference-in-differences (DID) model for causal identification. The specific model is as follows:


(1) $$\mathrm{EnvirPa}t_{ict}=\alpha_{0}+\alpha_{1}DID_{ct}+{X_{ict}}^{\prime }\theta +p_{i}+q_{t}+s_{\mathrm{pt}}+\varepsilon_{ict}$$

where *i, c*, *p*, and *t* represents the firm, city, province and year, respectively. *EnvirPat_ict_* denotes the number of green patents filed in year *t* by a listed company *i* within city *c*. *DID_ct_* equals one if city *c* is selected as a comprehensive demonstration city for the energy saving and emission reduction fiscal policy in year *t*, and zero otherwise. *X*_*ict*_ denotes a set of control variables. *p_i_* represents individual fixed effects. *q_t_* indicates time fixed effects. *s_pt_* represents joint fixed effects of provinces over time, and *ε_ict_* denotes error term.

In the baseline analysis, we focus on the coefficient of *DID_ct_* (*α*_1_), which reflects the impact of the energy saving and emission reduction fiscal policy on the green patent applications of firms within the experimental group of cities. If *α*_1_ is significantly positive, it means that the pilot policy helps to promote the green technology innovation of enterprises in the pilot area.

## Results and analyses

### Baseline regression results

Table 2 reports the regression results of the impact of the energy saving and emission reduction fiscal policy on corporate green technology innovation. We measure corporate green technology innovation using three indicators: the total number of green patent applications (*EnvirPat*), the number of green invention patent applications (*EnvirInvPat*), and the number of green utility model patent applications (*EnvirUtyPat*). In columns (1), (3), and (5), the results show that for *EnvirPat*, *EnvirInvPat*, and *EnvirUtyPat*, the coefficients of *DID* are all positive and significant at 1% level, indicating that the pilot policy promotes corporate green technology innovation. The models in columns (2), (4), and (6) further incorporate corporate control variables as well as province fixed effects over time. The results show that the coefficient of *DID* is significantly positive only when the dependent variable is *EnvirPat*. This indicates that additional regional factors do affect corporate green technology innovation, so it is necessary to control for all three fixed effects simultaneously in the baseline model, which can more accurately capture the impact of the pilot policy. After excluding possible confounding factors, the pilot policy results in a significant increase in the number of green patent applications. However, the impact of the pilot policy on green invention patents and green utility patents is not significant. The possible reason is that the primary task of the pilot policy is to reduce carbon in industries, thus the induced effect of the pilot policy on corporate green technology innovation is limited at the overall level without differentiating the carbon attributes of industries. This will be verified in the subsequent heterogeneity analyses in our paper.

**Table 2:** The impact of the energy saving and emission reduction fiscal policy on corporate green technology innovation.

	(1)	(2)	(3)	(4)	(5)	(6)
	*EnvirPat*	*EnvirPat*	*EnvirInvPat*	*EnvirInvPat*	*EnvirUtyPat*	*EnvirUtyPat*
*DID*	0.0705^***^	0.0386^**^	0.0563^***^	0.0222	0.0321^***^	0.0056
	(0.0144)	(0.0192)	(0.0119)	(0.0159)	(0.0103)	(0.0140)
*scale*		0.2346^***^		0.1943^***^		0.1443^***^
		(0.0066)		(0.0055)		(0.0048)
*lev*		0.0070		−0.0037		0.0151
		(0.0340)		(0.0280)		(0.0247)
*age*		−0.1114^***^		−0.0637^***^		−0.1034^***^
		(0.0199)		(0.0164)		(0.0145)
*fixasset*		−0.2343^***^		−0.2679^***^		−0.0611^*^
		(0.0463)		(0.0382)		(0.0337)
*share*		0.0163		−0.0026		0.0255
		(0.0309)		(0.0255)		(0.0225)
*tobinQ*		0.0802^***^		0.0764^***^		0.0460^***^
		(0.0164)		(0.0136)		(0.0120)
*one*		0.0368^***^		0.0335^***^		0.0291^***^
		(0.0128)		(0.0106)		(0.0093)
*Constant*	0.3044^***^	−4.5139^***^	0.2088^***^	−3.8440^***^	0.1795^***^	−2.7143^***^
	(0.0063)	(0.1562)	(0.0052)	(0.1288)	(0.0045)	(0.1138)
Individual	Yes	Yes	Yes	Yes	Yes	Yes
Year	Yes	Yes	Yes	Yes	Yes	Yes
Province × year	No	Yes	No	Yes	No	Yes
*R^2^*	0.0965	0.2104	0.0741	0.1902	0.0898	0.1801
*N*	16,873	15,716	16,873	15,716	16,873	15,716

***, **, and * denote statistical significance at the 1%, 5%, and 10% levels, respectively. T-statistics are based on robust standard errors and are presented in parentheses.

### Parallel trend test

The premise of using the DID method for policy evaluation is that the experimental and control groups need to satisfy parallel trends, which means that when the policy is not implemented, the trends of the green technology innovation of firms in the experimental cities and the control cities should remain parallel. To conduct a parallel trend test, we draw on Beck et al. (2010) and set up the following model:


(2) $$\begin{array}{c} \mathrm{EnvirPa}t_{ict}=\gamma +\overset{k=7}{\underset{k=-5}{\sum }}\beta_{k}\times D_{c,t+k}+{X_{\mathrm{it}}}^{\prime }\eta \\ +p_{i}+q_{t}+s_{\mathrm{pt}}+\varepsilon_{ict} \end{array}$$

In Equation (2), *D_c,t + k_* refers to a set of dummy variables that denote the *k*th year of policy implementation starting in city *c*. Since the pilot cities were implemented in three batches in 2011, 2013, and 2014, respectively, the sample interval covers the 5 years before and 7 years after implementation. $\beta_{k}$in the model is the coefficient we focus on and indicates the difference between the experimental and control groups at the *k*th year of the policy start. If none of the coefficient of $\beta_{k}$is significant in the period of *k < 0*, it demonstrates that the experimental and control groups satisfy the parallel trend assumption. If the coefficient of $\beta_{k}$in the period of *k < 0* are partially significant, it indicates that the experimental and control groups were significantly different before the policy was implemented and do not satisfy the common trend assumption.

**Figure 1:**
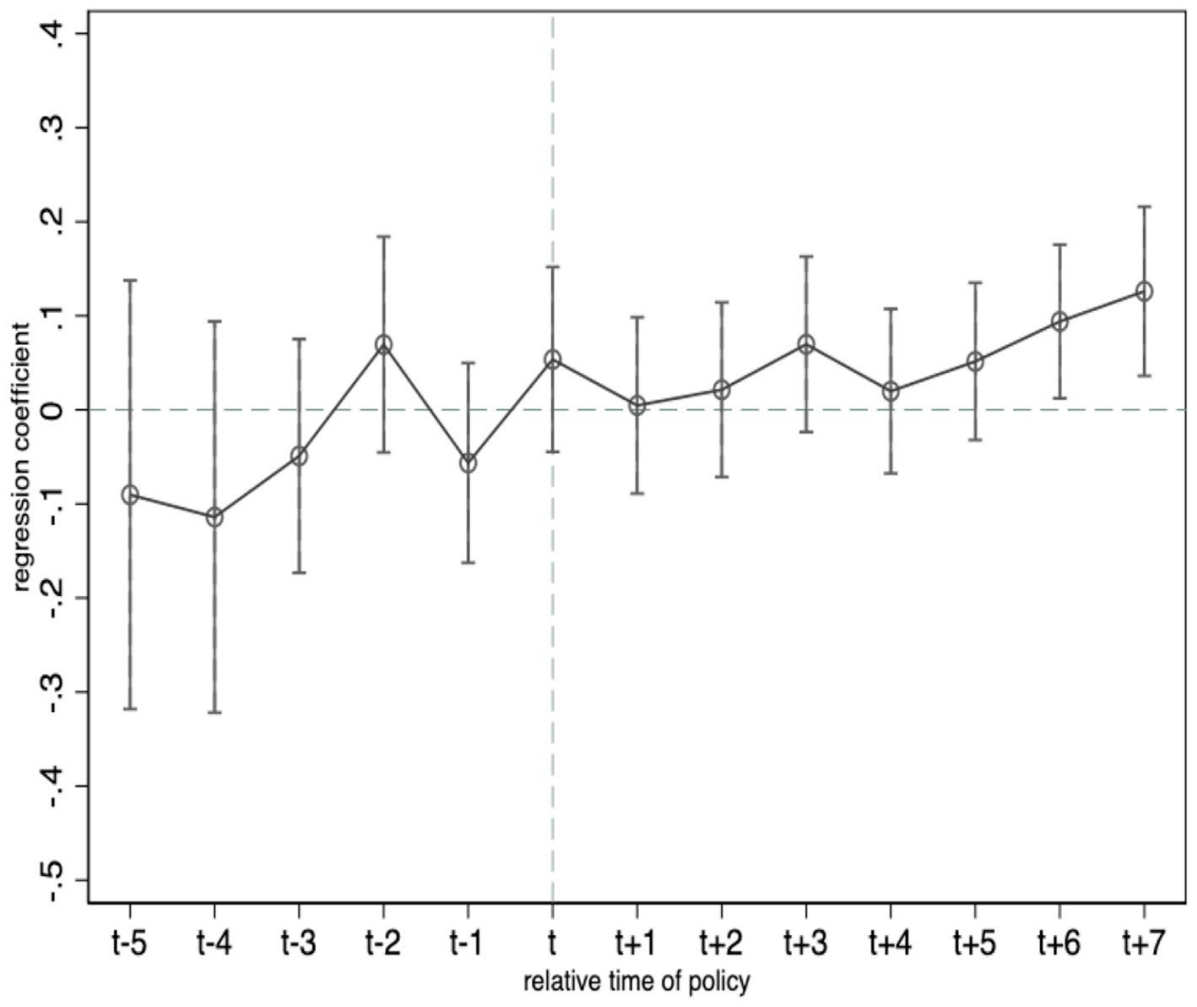
Parallel trend test.

Figure 1 illustrates the parallel trend test depicted with the total number of green patent applications as the dependent variable. We can see that, in the interval of *k < 0*, all estimated coefficients of $\beta_{k}$are not significant at the 95% confidence interval. And the values of the coefficients oscillate around 0, indicating that, prior to the implementation of the energy saving and emission reduction fiscal policy, there was no significant difference between the levels of the green technology innovation of firms in the experimental cities and the control cities. Starting from the fourth year after the policy’s implementation, firms within the experimental cities show an increase in green technology innovation year over year, indicating that there is at least a 3-year lag in the green technology innovation effect of the fiscal policy on energy conservation and emission reduction.

### Placebo test

Another concern regarding the identification assumption of the DID method is the interference of other unobservable city characteristics that change over time on the estimation results. There are differences across cities in terms of resource endowment, institutional environment, customs and culture, etc. Although the identification in the preceding section has taken province fixed effects over time into account, it is not possible to control for some time-varying, unobservable characteristics at the city level. To address this problem, we conduct an indirect placebo test which has been widely used in the relevant literature (Chetty et al., 2009). First, the expression of the coefficient can be derived from the following equation:


(3) $$\hat{\alpha_{1}}=\alpha_{1}+\delta \times \frac{cov\left(DID_{ct},\;\varepsilon_{ict}\;|W\right)}{var\left(DID_{ct}|W\right)}$$

where *W* includes all other control variables and fixed effects, and δ is the effect of unobservable factors on the dependent variable. If δ=0, then unobservable factors do not affect the estimation results. It means that $\hat{\alpha_{1}}$ is proved to be unbiased, but this cannot be directly verified. Therefore, we perform an indirect placebo test, the logic of which is to find an error variable that would theoretically have no effect on the outcome variable to take the place of *DID_ct_*. This variable is randomly generated. If this variable would actually have no effect on the results, then $\hat{\alpha_{1}}$=0. Conversely, then $\hat{\alpha_{1}}\neq 0$.

**Figure 2:**
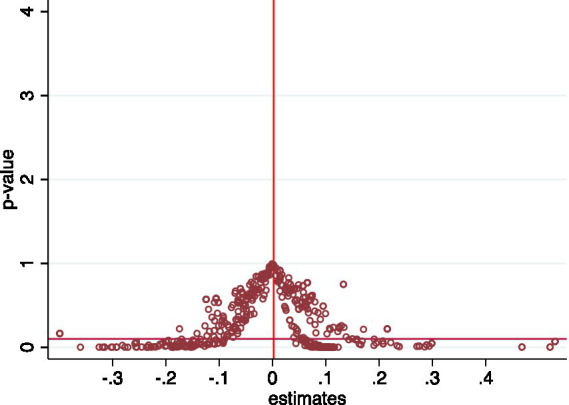
Placebo test.

Specifically, a “pseudo-policy dummy” is constructed by randomly selecting 27 cities out of 230 cities as the experimental group and the other cities as the control group, thereby generating a false coefficient of estimation. Since the “pseudo-experimental group” is randomly generated, the simulated policy dummy does not affect the dependent variable, and its wrong estimation coefficient should be close to 0. In our paper, 500 random samples are conducted. Figure 2 shows the significance and distribution of the estimated coefficients for the 500 random samples. The results show that the distribution of the “pseudo-policy dummy variables” is mostly concentrated around the zero point, and the corresponding *p*-values are higher than 0.1, consistent with the expectations of the placebo test.

### Other robustness tests

Firstly, we choose the number of green patents granted (*PatGrant*), the number of green invention patents granted (*InvPatGrant*) and the number of green utility patents granted (*UtyPatGrant*) as new dependent variables. The results in columns (1)–(3) in Table 3 show that, compared with the control group, the pilot policy leads to a significant increase in the number of green patents granted to the treatment group, among which, the impact on the number of green invention patents granted is significant, and the impact on the number of green utility patents granted is not significant.

**Table 3:** Robustness tests: replacing core variables.

	(1)	(2)	(3)	(4)	(5)	(6)
	*PatGrant*	*InvPatGrant*	*UtyPatGrant*	*EnvirPat*	*EnvirInvPat*	*EnvirUtyPat*
*DID*	0.0453^**^	0.0324^***^	0.0040	0.0419^**^	0.0264^*^	0.0070
	(0.0184)	(0.0122)	(0.0158)	(0.0194)	(0.0160)	(0.0141)
*Control variables*	Yes	Yes	Yes	Yes	Yes	Yes
*Individual*	Yes	Yes	Yes	Yes	Yes	Yes
*Year*	Yes	Yes	Yes	Yes	Yes	Yes
*Province × year*	Yes	Yes	Yes	Yes	Yes	Yes
*R^2^*	0.2032	0.1430	0.1835	0.2104	0.1903	0.1801
*N*	13,794	13,794	13,794	15,716	15,716	15,716

***, **, and * denote statistical significance at the 1%, 5%, and 10% levels, respectively. T-statistics are based on robust standard errors and are presented in parentheses.

Secondly, as the time of setting up the second batch of comprehensive demonstration cities for the energy saving and emission reduction fiscal policy was October 2013 and there may be a certain time lag from the introduction of the national policy to the implementation by local governments, the robustness test takes 2014 as the policy shock node for the second batch of pilot cities. The results in columns (4)–(6) in Table 3 show that the pilot policy still promotes corporate green patent applications at the 5% significance level and promotes corporate green invention patent applications at the 10% significance level, but the effect on green utility patent applications is insignificant.

Thirdly, in order to avoid potential sample self-selection problems, we further adopt the PSM-DID method for robustness testing. Since our sample size is sufficient, we choose a more stringent caliper radius (0.0001) for matching in order to minimize the impact of “selectivity bias.” The results in columns (1)–(3) in Table 4 show that the energy conservation and emission reduction fiscal policy still promotes firms’ green patent applications at the 5% significance level, which remains consistent with the baseline results. In addition, due to the fact that the PSM method is highly dependent on the formal setting of the first-stage logistic model, Hainmueller (2012) proposes an Entropy Balancing method that does not depend on the setting of the first-stage logistic model. Therefore, we also use the Entropy Balancing method to conduct our robustness test. Columns (4)–(6) in Table 4 report the difference-in-differences estimates after matching through the Entropy Balancing method. The results show that the pilot policy can still significantly promote corporate green technology innovation, which further strengthens the robustness of the baseline results.

**Table 4:** Robustness tests: Reducing selection bias.

	(1)	(2)	(3)	(4)	(5)	(6)
	*EnvirPat*	*EnvirInvPat*	*EnvirUtyPat*	*EnvirPat*	*EnvirInvPat*	*EnvirUtyPat*
*DID*	0.0396^**^	0.0227	0.0063	0.0386^**^	0.0222	0.0056
	(0.0192)	(0.0159)	(0.0140)	(0.0192)	(0.0159)	(0.0140)
Control variables	Yes	Yes	Yes	Yes	Yes	Yes
Individual	Yes	Yes	Yes	Yes	Yes	Yes
Year	Yes	Yes	Yes	Yes	Yes	Yes
Province × year	Yes	Yes	Yes	Yes	Yes	Yes
*R^2^*	0.2108	0.1906	0.1803	0.2104	0.1902	0.1801
*N*	15,707	15,707	15,707	15,716	15,716	15,716

***, ** and * denote statistical significance at the 1%, 5%, and 10% levels, respectively. T-statistics are based on robust standard errors and are presented in parentheses.

Finally, during the period of model city construction, some pilot cities may also be stimulated by other innovative policies, such as the low-carbon city pilot policy initiated by the National Development and Reform Commission (NDRC) in 2010. It has been shown that the low-carbon city pilot policy can significantly induce green technology innovation among high-carbon emitting firms (Xu and Cui, 2020). In addition, in October 2011, the NDRC issued the Notice on Piloting Carbon Emissions Trading, which formally approved seven provinces and cities to carry out pilot carbon trading. Such policies may also promote green technology innovation among enterprises. In order to exclude the interference of other confounding policies at the city level, we control for the above two types of policies separately. The results in Table 5 show that after controlling for the interference of other policies in the same period, the energy saving and emission reduction fiscal policy still significantly promotes the green patent applications of enterprises.

**Table 5:** Robustness tests: excluding confounding policies.

	(1)	(2)	(3)	(4)	(5)	(6)
	*EnvirPat*	*EnvirInvPat*	*EnvirUtyPat*	*EnvirPat*	*EnvirInvPat*	*EnvirUtyPat*
*DID*	0.0386^**^	0.0222	0.0056	0.0370^*^	0.0200	0.0061
	(0.0192)	(0.0159)	(0.0140)	(0.0195)	(0.0161)	(0.0142)
Carbon emissions trading	Yes	Yes	Yes	Yes	Yes	Yes
Low carbon city pilot				Yes	Yes	Yes
Control variables	Yes	Yes	Yes	Yes	Yes	Yes
Individual	Yes	Yes	Yes	Yes	Yes	Yes
Year	Yes	Yes	Yes	Yes	Yes	Yes
Province × year	Yes	Yes	Yes	Yes	Yes	Yes
*R^2^*	0.2104	0.1902	0.1801	0.2104	0.1902	0.1801
*N*	15,716	15,716	15,716	15,716	15,716	15,716

^***^, ^**^, and ^*^ denote statistical significance at the 1%, 5%, and 10% levels, respectively. T-statistics are based on robust standard errors and are presented in parentheses.

## Additional analyses

### Mechanism analyses

The above analyses show that the fiscal policy for energy efficiency and emission reduction can promote corporate green technology innovation, so what is the inherent transmission mechanism? In order to financially assist the transformation of the pilot cities to low-carbon cities, green fiscal policies have been implemented in these cities. For enterprises facing financial constraints within pilot cities, the relevant financial departments will provide them with incentive funds like credit support and government subsidies to ease their financial pressure during the process of technological transformation. We construct a difference-in-differences-in-differences (DDD) model based on equation (1) to examine the credit mechanism and the government subsidy mechanism generated by the pilot policy. The specific models are as follows:


(4) $$\begin{array}{l} \mathrm{EnvirPa}t_{ict}=\gamma_{0}+\gamma_{1}\mathrm{trea}t_{ct}\times time_{ct}\times loan_{ct}\\ +\gamma_{2}\mathrm{trea}t_{ct}\times loan_{ct}+\gamma_{3}time_{ct}\times loan_{ct}\\ +\gamma_{4}\mathrm{trea}t_{ct}\times time_{ct}+{X_{ict}}^{\prime }\theta +p_{i}+q_{t}+s_{\mathrm{pt}}+\varepsilon_{ict} \end{array}$$

(5) $$\begin{array}{l} \mathrm{EnvirPa}t_{ict}=\gamma_{0}+\gamma_{1}\mathrm{trea}t_{ct}\times time_{ct}\times sub_{\mathrm{it}}+\gamma_{2}\mathrm{trea}t_{ct}\\ \times \;sub_{\mathrm{it}}+\gamma_{3}time_{ct}\times sub_{\mathrm{it}}+\gamma_{4}\mathrm{trea}t_{ct}\times time_{ct}\\ +{X_{ict}}^{\prime }\zeta +p_{i}+q_{t}+s_{\mathrm{pt}}+\varepsilon_{ict} \end{array}$$

In equation (4), (5), *treat_ct_* denotes the policy grouping dummy variable, *time_ct_* denotes the policy time dummy variable, *loan_ct_* denotes the logarithm of the balance of all loans from financial institutions in the city *c* at the end of year *t,* and *sub_it_* denotes the government subsidies received by firm *i* in year *t*. *sub_it_* is computed as the natural logarithm of one plus the government subsidies received by enterprises. The results are shown in Table 6.

**Table 6:** Credit and Government Subsidy Mechanism Inspection.

	(1)	(2)	(3)	(4)	(5)	(6)
	*EnvirPat*	*EnvirInvPat*	*EnvirUtyPat*	*EnvirPat*	*EnvirInvPat*	*EnvirUtyPat*
*treat × time*	−0.7320^***^	−0.6306^***^	−0.2898	−0.7964^***^	−0.7525^***^	−0.4403^***^
	(0.2620)	(0.2161)	(0.1909)	(0.1175)	(0.0966)	(0.0859)
*treat × loan*	−0.0013	−0.0017	−0.0012			
	(0.0022)	(0.0018)	(0.0016)			
*treat × time × loan*	0.0425^***^	0.0366^***^	0.0170^*^			
	(0.0141)	(0.0117)	(0.0103)			
*treat × sub*				−0.0012	−0.0017	−0.0012
				(0.0023)	(0.0019)	(0.0017)
*treat × time × sub*				0.0524^***^	0.0491^***^	0.0284^***^
				(0.0075)	(0.0061)	(0.0055)
Control variables	Yes	Yes	Yes	Yes	Yes	Yes
Individual	Yes	Yes	Yes	Yes	Yes	Yes
Year	Yes	Yes	Yes	Yes	Yes	Yes
Province × year	Yes	Yes	Yes	Yes	Yes	Yes
*R^2^*	0.2112	0.1911	0.1804	0.2115	0.1924	0.1810
*N*	15,698	15,698	15,698	15,129	15,129	15,129

***, **, and * denote statistical significance at the 1%, 5%, and 10% levels, respectively. T-statistics are based on robust standard errors and are presented in parentheses.

Columns (1)–(3) of Table 6 show the results of the credit mechanism test. It can be found that the coefficients of the interaction (*treat* × *time* × *loan*) are significantly positive, which indicates that the energy saving and emission reduction fiscal policy can promote corporate green technology innovation by increasing the overall loan balance at the city level. Columns (4)–(6) of Table 6 report the results of the government subsidy mechanism. The results show that the coefficients of the interaction (*treat* × *time* × *sub*) are all significantly positive at the 1% level, indicating that the pilot policy can promote enterprises’ green technology innovation through the government subsidy mechanism. The results together suggest that the pilot policy does alleviate the psychological barriers faced by the management in making organizational change (i.e., corporate green innovation) through the credit mechanism and the government subsidy mechanism, which enriches the literature on organizational psychology and organizational change from the perspective of corporate green innovation.

Another issue is whether enterprises in the demonstration cities decide to increase their R&D expenditures after getting credit support and government subsidies. It has been pointed out that after receiving credit support or government subsidies, firms are likely to use the funds either to increase R&D investment or to expand fixed asset investment (Qian et al., 2021). Therefore, we further test the innovation input mechanism generated by the pilot policy, which is modeled as follows:


(6) $$lnrd_{ict}=d_{0}+d_{1}DID_{ct}+{X_{ict}}^{\prime }\rho +p_{i}+q_{t}+s_{\mathrm{pt}}+\varepsilon_{ict}$$

(7) $$\mathrm{tensit}y_{ict}=f_{0}+f_{1}DID_{ct}+{X_{ict}}^{\prime }\varphi +p_{i}+q_{t}+s_{\mathrm{pt}}+\varepsilon_{ict}$$

In Equations (6), (7), *Inrd_ict_* denotes the logarithm of the R&D expenditure of firm *i* in year *t*, and *tensity_ict_* denotes the R&D intensity of firm *i* in year *t*. Here we use the ratio of the R&D expenditure of firm *i* to sales revenue to measure *tensity_ict_*. Table 7 reports the results of the innovation input mechanism of the pilot policy. It can be found that the pilot policy increases the R&D intensity of firms, indicating that the energy saving and emission reduction fiscal policy promotes firms’ green technology innovation through innovation compensation effect. Thus, hypothesis 2a is verified.

**Table 7:** Innovation input mechanism test.

	(1)	(2)
	*lnrd*	*tensity*
*DID*	0.1313	0.0053^***^
	(0.2184)	(0.0016)
*Control variables*	Yes	Yes
*Individual*	Yes	Yes
*Year*	Yes	Yes
*Province × year*	Yes	Yes
*R^2^*	0.4928	0.1742
*N*	10,122	10,122

***, **, and * denote statistical significance at the 1%, 5%, and 10% levels, respectively. T-statistics are based on robust standard errors and are presented in parentheses.

### Heterogeneity analyses

#### Heterogeneity of enterprise

Different ownership types usually have different impacts on firms’ R&D investment and technological innovation. To test whether ownership type will influence the effect of the pilot policy on corporate green technology innovation, we split the whole sample into two subsamples based on firms’ ownership type, and re-estimate model (1) separately.

The results in Table 8 show that the pilot policy significantly promotes the green technology innovation of non-SOEs. The possible reason is that non-SOEs usually face credit discrimination in the capital market, and the energy conservation and emission reduction fiscal policy makes non-SOEs have stronger incentives to choose green technology innovation through the support of green special funds. On the contrary, SOEs are invested or controlled by the central government or local governments and have a great advantage in resource allocation, especially in obtaining financial support (Allen et al., 2005). With their political power, SOEs can not only acquire more financial funds, but also reduce the environmental pressure from regulations. Therefore, when faced with environmental constraints, SOEs are more likely to develop “innovation inertia.”

**Table 8:** The heterogeneity of enterprise ownership.

	*EnvirPat*		*EnvirInvPat*		*EnvirUtyPat*	
	*SOEs*	*Non-SOEs*	*SOEs*	*Non-SOEs*	*SOEs*	*Non-SOEs*
*DID*	−0.1138^**^	0.0837^***^	−0.1235^***^	0.0689^***^	−0.0681^*^	0.0250
	(0.0505)	(0.0216)	(0.0429)	(0.0174)	(0.0363)	(0.0160)
*Control variables*	Yes	Yes	Yes	Yes	Yes	Yes
*Individual*	Yes	Yes	Yes	Yes	Yes	Yes
*Year*	Yes	Yes	Yes	Yes	Yes	Yes
*Province × year*	Yes	Yes	Yes	Yes	Yes	Yes
*R^2^*	0.2751	0.2002	0.2544	0.1776	0.2531	0.1752
*N*	4,868	10,159	4,868	10,159	4,868	10,159

***, **, and * denote statistical significance at the 1%, 5%, and 10% levels, respectively. T-statistics are based on robust standard errors and are presented in parentheses.

Further, we also examine the impact of enterprise life cycle on the association between the pilot policy and corporate green technology innovation. We refer to the cash flow model of Dickinson (2011) and Liu et al. (2020) to classify the enterprise life cycle. The positive and negative combinations of three cash flow types (i.e., net cash flow from operating activities, net cash flow from investing activities, and net cash flow from financing activities) are used to reflect the business risk, profitability, and growth rate of enterprises in different life cycles.

The findings in Table 9 demonstrate that after controlling for all three fixed effects simultaneously, the pilot policy has a significant effect on the green technology innovation of mature firms, regardless of whether green patent applications or green invention patent applications are used as the dependent variable. The pilot policy also plays a role in promoting the green patent applications of growth firms, while it does not have any significant effect on the green technology innovation of declining firms. The reason may be as follows. On the one hand, growth firms are full of innovative energy but have insufficient R&D experience, so they are less likely to make successful green invention innovation which has higher technical difficulty. On the other hand, mature firms are becoming more sophisticated in their production model, constantly updating their organizational structure, and having a wide range of partners and interest groups in the market, so they have sufficient funds to purchase advanced emission equipment and carbon reduction facilities, and tend to invest in green innovation, especially in green invention innovation with greater innovation breakthrough and higher future income. However, declining firms are more likely to suffer from institutional rigidity, section redundancy and worsening financial conditions, which leads them to become more conservative and unwilling to invest in green innovation which is characterized by high investment, high cost and high risk (Kueng et al., 2014).

**Table 9:** The heterogeneity of enterprise life cycle.

	*EnvirPat*			*EnvirInvPat*			*EnvirUtyPat*		
	*Growth*	*Maturity*	*Decline*	*Growth*	*Maturity*	*Decline*	*Growth*	*Maturity*	*Decline*
*DID*	0.0636^**^	0.0961^**^	−0.0325	0.0345	0.0830^**^	−0.0361	0.0313	−0.0379	0.0193
	(0.0293)	(0.0439)	(0.0328)	(0.0240)	(0.0357)	(0.0275)	(0.0214)	(0.0239)	(0.0312)
*Control variables*	Yes	Yes	Yes	Yes	Yes	Yes	Yes	Yes	Yes
*Individual*	Yes	Yes	Yes	Yes	Yes	Yes	Yes	Yes	Yes
*Year*	Yes	Yes	Yes	Yes	Yes	Yes	Yes	Yes	Yes
*Province × year*	Yes	Yes	Yes	Yes	Yes	Yes	Yes	Yes	Yes
*R^2^*	0.2119	0.2748	0.2654	0.1978	0.2468	0.2466	0.1861	0.2389	0.2496
*N*	7,547	5,537	2,547	7,547	5,537	2,547	7,547	5,537	2,547

***, **, and * denote statistical significance at the 1%, 5%, and 10% levels, respectively. T-statistics are based on robust standard errors and are presented in parentheses.

#### Heterogeneity of industry

The tasks of the energy saving and emission reduction fiscal policy include six aspects, the main one of which is to achieve industrial low-carbonization. The policy may be more beneficial for high-carbon industries because it places great emphasis on the low-carbonization of industrial structures. To examine whether the impact of the pilot policy on corporate green technology innovation differs between high-carbon and low-carbon industries, we identify six industries as high-carbon industries^2^ according to the Report on China’s Carbon Emission Trading Report (2017). Table 10 reports the test results of the group regressions.

**Table 10:** The heterogeneity of industry.

	EnvirPat		EnvirInvPat		EnvirUtyPat	
	High carbon industries	Low carbon industries	High carbon industries	Low carbon industries	High carbon industries	Low carbon industries
*DID*	0.1153^***^	0.0192	0.1124^***^	0.0021	−0.0131	0.0069
	(0.0407)	(0.0221)	(0.0350)	(0.0181)	(0.0253)	(0.0165)
Control variables	Yes	Yes	Yes	Yes	Yes	Yes
Individual	Yes	Yes	Yes	Yes	Yes	Yes
Year	Yes	Yes	Yes	Yes	Yes	Yes
Province × year	Yes	Yes	Yes	Yes	Yes	Yes
*R^2^*	0.2043	0.2321	0.1686	0.2165	0.2406	0.1920
*N*	3,531	12,152	3,531	12,152	3,531	12,152

***, **, and * denote statistical significance at the 1%, 5%, and 10% levels, respectively. T-statistics are based on robust standard errors and are presented in parentheses.

The results in Table 10 show that after controlling for the three fixed effects simultaneously, the pilot policy significantly promotes the green patent applications as well as green invention patent applications of firms in high-carbon industries, but it has no significant effect on the green technology innovation of firms in low-carbon industries. From the above analyses, it can be seen that there is significant industrial heterogeneity in the impact of the energy saving and emission reduction fiscal policy on corporate green technology innovation, and that the positive effect of the pilot policy on enterprises’ green technology innovation in high-carbon industries is more significant than that in low-carbon industries.

## Conclusions and policy Implications

### Conclusions

Green technology innovation is an important force to promote the low-carbon development of industries. Using data from Chinese A-share listed firms in manufacturing industry from 2008 to 2019, we examine whether and how the energy saving and emission reduction fiscal policy affects firms’ green technology innovation. Our paper finds that, firstly, the pilot policy significantly promotes corporate green technology innovation. This finding remains after we conduct a series of robustness checks, including placebo tests, using alternative measures of green technology innovation, and excluding confounding policies. Secondly, the effect of the pilot policy on corporate green technology innovation is heterogeneous at firm-level and industry-level. At the firm level, the positive effect of the pilot policy on green technology innovation is more pronounced in non-state-owned enterprises and mature enterprises. At the industry level, the pilot policy is more helpful to promote the green technology innovation of firms in high-carbon industries. Thirdly, credit support, government subsidies and enhanced innovation inputs are the key channels through which the pilot policy promotes corporate green technology innovation.

### Policy implications

Our findings provide the following policy implications for effectively promoting the construction of comprehensive demonstration cities for energy saving and emission reduction fiscal policy and encouraging corporate green technology innovation.

Firstly, the scope and areas of demonstration work on the energy saving and emission reduction fiscal policy should be further expanded to promote the green and low-carbon transformation of enterprises in the pilot cities. Our research demonstrates that the energy saving and emission reduction fiscal policy do have a positive impact on the green technological innovation of firms. Therefore, local governments need to summarize and refine the pilot experience in a timely manner, form experience models, and thus provide experience and reference for the achievement of China’s emission peak and carbon neutrality targets from the city level. At the same time, in the process of using financial funds to guide corporate green technology innovation, pilot cities should be effectively supervised and guided in order to fully induce corporate green technology progress and thus achieve a win-win situation of low-carbon emission reduction and economic development.

Secondly, the transition of high-carbon industries to low-carbon direction is an important source of corporate green technology innovation. A clearer technology transition guidance program should be developed for high-carbon industries to further induce more innovative green invention innovation. The industry heterogeneity test shows that the pilot policy promotes green technology innovation more significantly in high-carbon industries than in low-carbon industries, and that the green technology innovation in high-carbon industries is mainly manifested in green invention patents. This indicates that the transformation of high-carbon industries plays an important role in promoting the low-carbonization of industries in the pilot cities, and that the higher-value green invention patents are the key to promote the transformation of high-carbon industries to low-carbonization. Therefore, to improve the effectiveness of the pilot program, local governments should continue to use the policy to encourage high-carbon industries to conduct more green technology innovation and achieve low-carbon transformation.

## Data availability statement

The raw data supporting the conclusions of this article will be made available by the authors, without undue reservation.

## Author contributions

YC: integrated arrangement, writing of the first draft, theoretical analysis, revision checking, and funding support. HJ: data processing, theoretical analysis, writing of the first draft, and revision checking. JY: literature search and organization, data collection and organization, and data processing. All authors contributed to the article and approved the submitted version.

## Funding

YC acknowledges financial support from the National Natural Science Foundation of China (grant no. 72102024), China Postdoctoral Science Foundation (grant no. 2021M700575), and the Fundamental Research Funds for the Central Universities (project no. 2021CDSKXYJG007).

## Conflict of interest

The authors declare that the research was conducted in the absence of any commercial or financial relationships that could be construed as a potential conflict of interest.

## Publisher’s note

All claims expressed in this article are solely those of the authors and do not necessarily represent those of their affiliated organizations, or those of the publisher, the editors and the reviewers. Any product that may be evaluated in this article, or claim that may be made by its manufacturer, is not guaranteed or endorsed by the publisher.

## Footnotes

^1^Note: In order to avoid the influence of zero value, the number of the three kinds of patent applications are processed by adding one and then taking the logarithm.

^2^Note: The specific high carbon emission industries in the text include: paper and paper products industry (C22), petroleum processing, coking and nuclear fuel processing industry (C25), chemical raw materials and chemical products manufacturing industry (C26), non-metallic mineral products industry (C30), ferrous metal smelting and rolling processing industry (C31), non-ferrous metal smelting and rolling processing industry (C32).
